# Isolated Tuberculosis of the Wrist: Subtle but Destructive

**DOI:** 10.7759/cureus.7939

**Published:** 2020-05-03

**Authors:** Ammar Manas, Mohd Yazid Bajuri, Rashdeen Fazwi Muhammad Nawawi

**Affiliations:** 1 Orthopaedic, Hospital Kuala Lumpur, Kuala Lumpur, MYS; 2 Orthopaedics and Traumatology, Universiti Kebangsaan Malaysia, Kuala Lumpur, MYS; 3 Orthopaedics, Hospital Kuala Lumpur, Kuala Lumpur, MYS

**Keywords:** wrist tuberculosis, extrapulmonary tuberculosis, osteolysis, anti-tuberculosis, osteomyelitis

## Abstract

Tuberculosis is an infection that can occur in every organ of the body, but it rarely affects the wrist joint. We report a rare case of a male patient with wrist tuberculosis with a subtle presentation. Our patient’s left wrist had been swollen for four months and progressively worsened, becoming ulcerated one week prior to presentation to our center. He was asymptomatic, but a previous radiograph showed global destruction of the wrist joint. Clinical investigations, that is, polymerase chain reaction test for tuberculosis and histopathological examination, showed classic findings of tuberculosis, which lead to the initiation of anti-tuberculosis treatment.

## Introduction

Tuberculosis is a major source of morbidity and mortality, especially in Africa and parts of Asia [1]. The incidence of tuberculosis is increasing worldwide due to increased immigration and immunosuppression [1]. The infection is caused by Mycobacterium tuberculosis and could affect virtually any organ in the body. Although it involves the pulmonary system primarily, extrapulmonary tuberculosis is also possible.

## Case presentation

**Figure 1:**
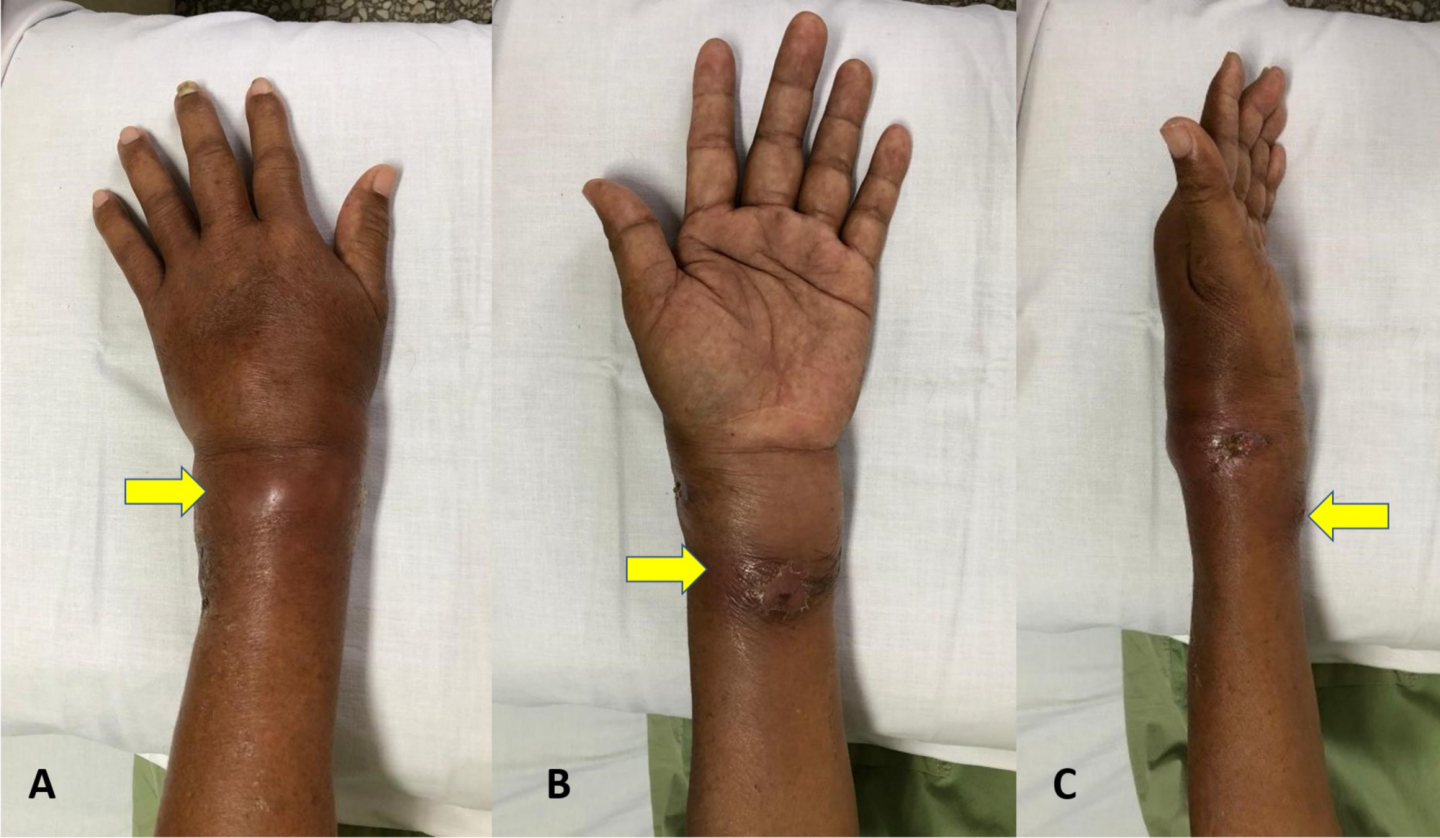
Swelling of the wrist from the dorsal (A), volar (B), and lateral (C) aspects.

A 58-year-old right hand-dominant contractor originally from Indonesia with no known comorbidities presented to our clinic. He had been suffering from left wrist swelling for four months, with the swelling gradually increasing in size. Initially, the swelling started at the dorsum of the wrist and was of the size of a marble (1 cm x 1 cm); it gradually increased in size and involved the volar aspect of the wrist, reaching the size of a tennis ball (5 cm x 5 cm). The swelling was associated with minimal pain on motion and occasional nocturnal fever. Over the week prior to presentation, the swollen tissue had become ulcerated and had a purulent discharge. The patient denied any history of trauma, animal bite, weight or appetite loss, or swelling elsewhere. On examination, we noted diffuse edema of the wrist involving the volar and dorsal aspects, measuring 9 cm x 7 cm with fluctuance (especially over the dorsum of the wrist), warmth, and erythema with minimal tenderness (Figure 1). There was a punctum measuring 0.5 cm x 0.5 cm at the radial side of the wrist, as well as seropurulent discharge. The wrist motion limited to 0-15 degrees for both flexion and extension of the wrist, with minimal numbness over the volar aspect of the right hand. The swelling was non-pulsatile, and there was no other swelling noted elsewhere.

**Figure 2:**
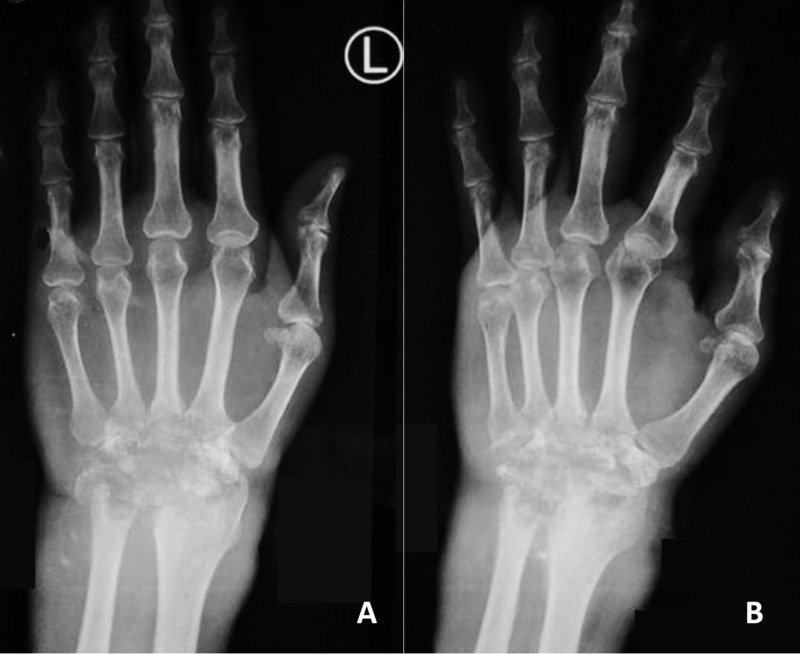
Plain radiograph of the wrist. (A) Anteroposterior view. (B) Oblique view.

On plain radiography, global destruction and lytic lesions of the carpal bones were noted at the distal ends of the radius and ulnar and at the base of the second to fifth metacarpal bones (Figure 2).

Complete blood count showed healthy values with a white blood cell count of 6.1 x 109/L, hemoglobin of 11.8 g/dL, and a platelet count at 235 x 109/L. The erythrocyte sedimentation rate was elevated at 74 mm/hour, and C-reactive protein was 29.4 g/dL. Viral screens for HIV, hepatitis, and syphilis were negative. Biochemical parameters such as renal function, liver function, glycated hemoglobin, and uric acid level were within reference ranges. Mantoux test was positive at 15 mm. Plain radiography of the chest was unremarkable.

Ultrasound imaging of the swelling revealed a multiloculated collection with intra-articular extension. At this point, the patient was treated for wrist abscess and osteomyelitis of the carpal bones, with tuberculosis of the wrist being on the differential diagnosis.

**Figure 3:**
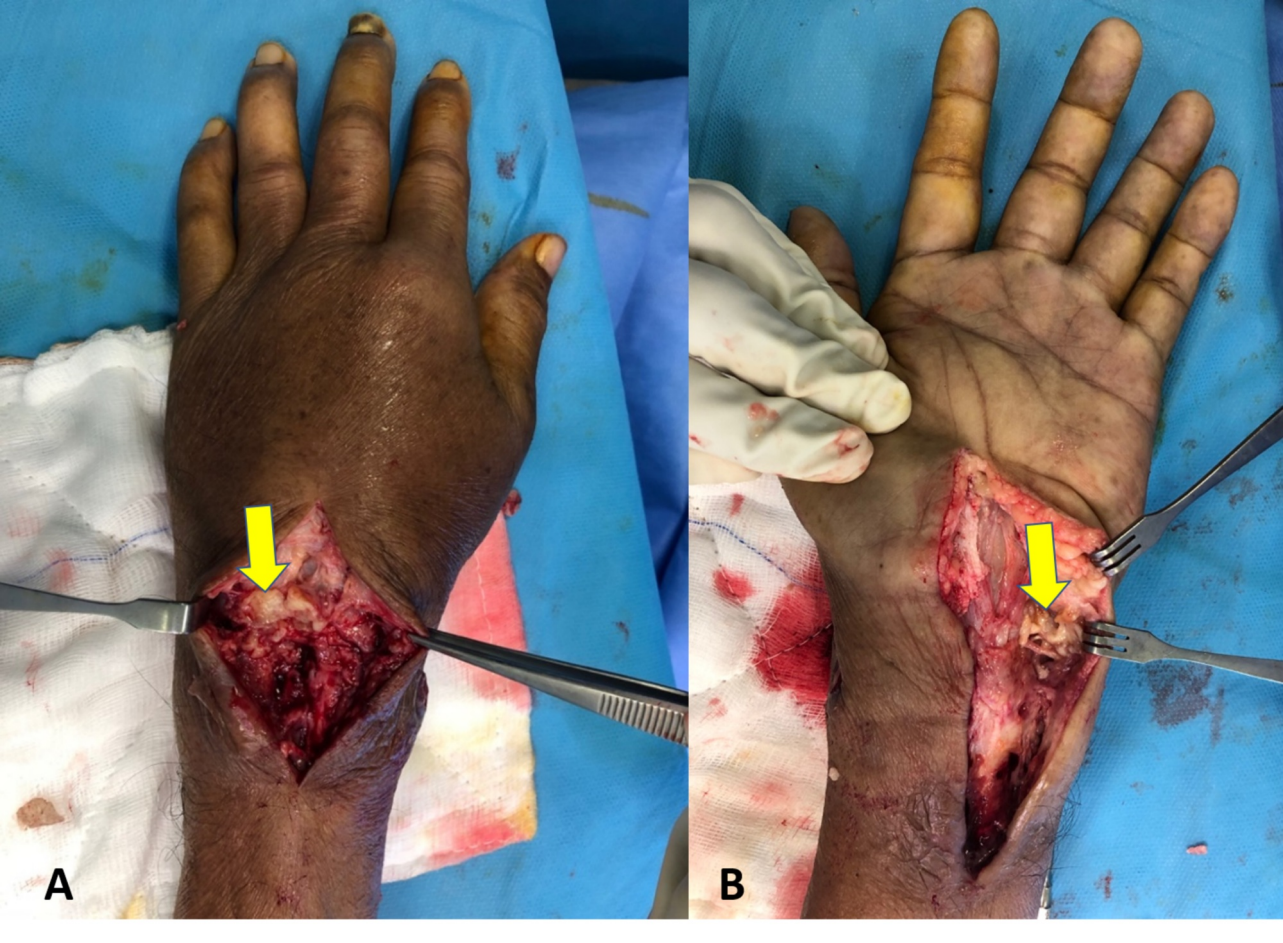
Intraoperative picture showing cheese-like material from the dorsal (A) and volar (B) aspects of the wrist.

**Figure 4:**
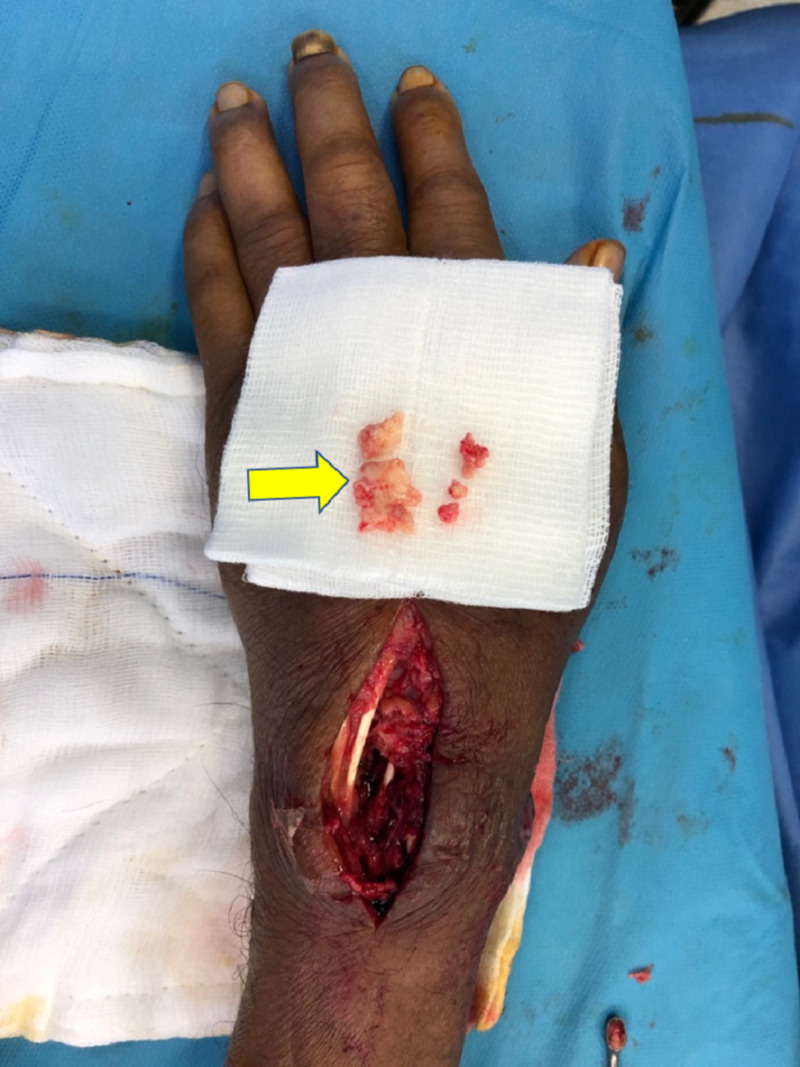
Intraoperative picture showing a fragmented carpal bone.

Under general anesthesia, debridement and curettage of the left wrist were performed. Intraoperatively, minimal pus was noted over the dorsal aspect of the left wrist between the third and fourth dorsal compartments with cheese-like material underneath the extensor retinaculum communicating with the carpal bones (Figure 3). The purulent discharge was connected to the volar aspect of the subcutaneous layer at the ulnar border and involved the carpal tunnel. Carpal tunnel release was also performed for the eradication of necrotic tissue in view of the extension of the infection. The carpal bones were eroded and fragmented (Figure 4). The wrist was immobilized using a thermoplastic splint postoperatively.

**Figure 5:**
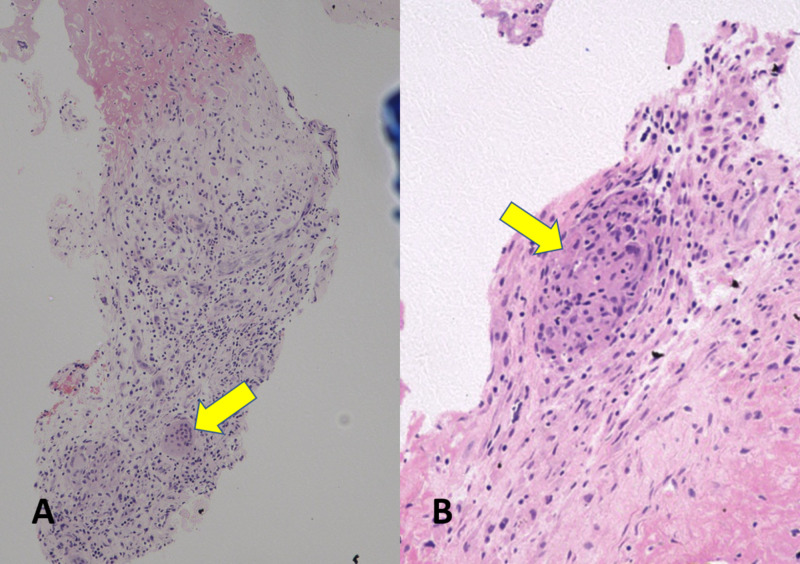
Histological examination showing necrotic bone with granulation and giant cell reaction noted, with infiltration of histiocytes, neutrophils, lymphocytes, and plasma cells consistent with chronic granulomatous inflammation. (A) 10x magnification. (B) 20x magnification.

A sample was sent for culture and sensitivity, histopathological examination, and acid-fast bacteria (AFB) culture. Results were negative for AFB culture. Based on histopathological examination, an epithelioid granulomatous reaction with central necrosis was identified, suggestive of tuberculosis (Figure 5). The results of a polymerase chain reaction test for tuberculosis were positive for tuberculosis. The patient was diagnosed with tuberculosis of the wrist and was started on anti-tuberculosis treatment.

**Figure 6:**
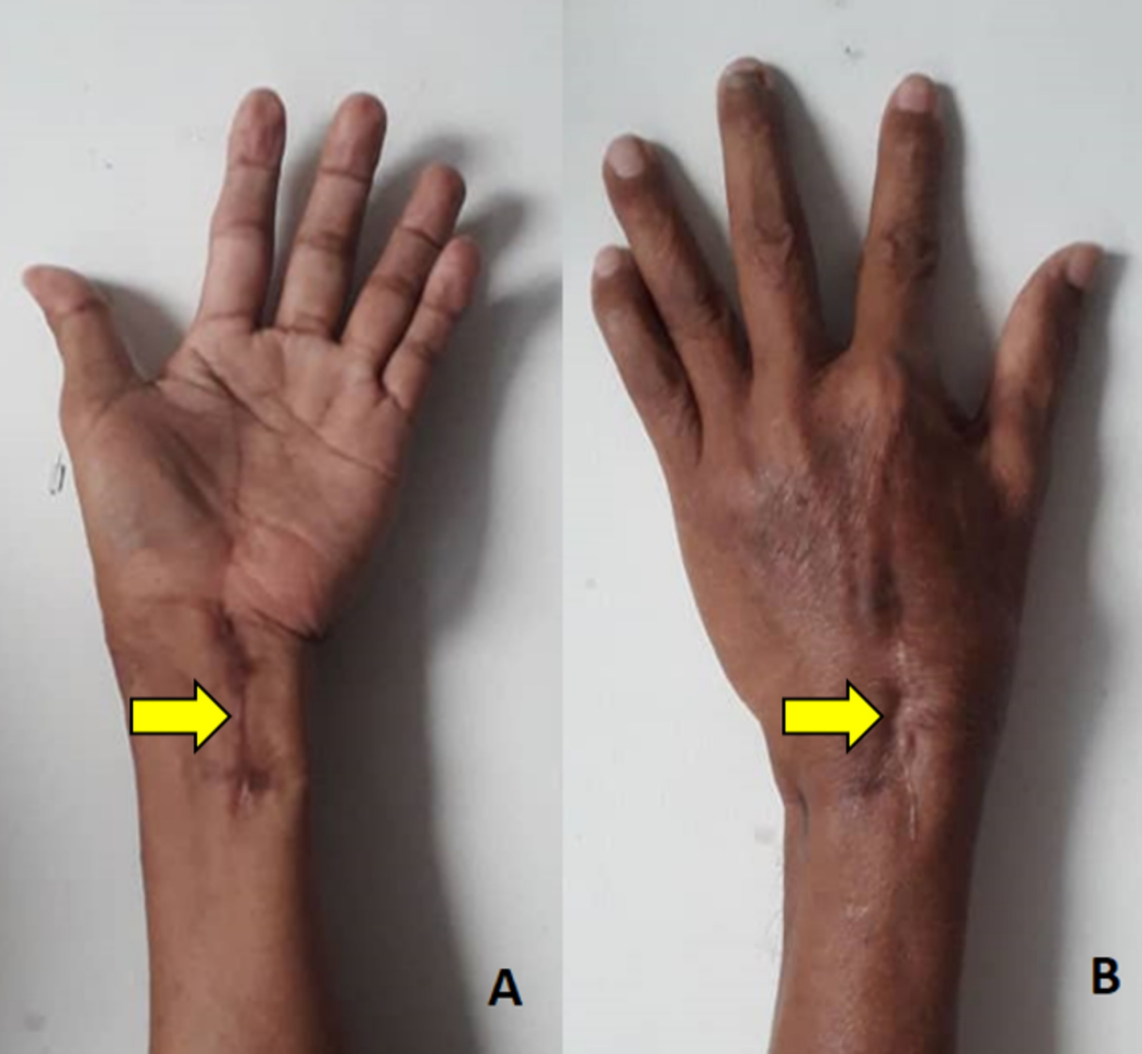
Clinical picture of the left wrist after one year. (A) Volar aspect of the wrist. (B) Dorsal aspect of the wrist.

Upon follow-up at six weeks, the wound was completely healed and he was able to resume his normal activity. Subsequently, he resumed work at three months postoperatively. He never misses his medication and had been compliant with the anti-tuberculosis therapy for 1 year. At 1-year follow-up, the swelling has subsided (Figure 6), range of motion was normal, and, ultimately, he was satisfied with the functional outcome of the wrist.

## Discussion

Bone is the third most common site of tuberculosis after the lungs and lymph nodes, accounting for around 10% of all extrapulmonary tuberculosis [1]. Tuberculosis mostly affects large weight-bearing joints such as those in the spine, hip, and knee. Nevertheless, nearly 1% of extrapulmonary tuberculosis has been described in wrist joints [1]. Because of the rarity of wrist tuberculosis, making a diagnosis is often difficult during the initial encounter. Sivasamy et al. reported that extrapulmonary tuberculosis is difficult to diagnose as it has multiple possible differential diagnoses such as subacute or chronic suppurative arthritis, osteochondrosis, rheumatoid arthritis, Kaposi sarcoma, and benign bone tumors [2].

Usually, patients have nonspecific symptoms such as joint pain, swelling, stiffness, and wound discharge. They also have systemic manifestations such as fever, night sweats, and weight loss [3].

The erythrocyte sedimentation rate is almost always elevated. However, in atypical tuberculosis, the value can be normal [4]. The presence of acid-fast bacilli on Ziehl-Neelsen staining may help in diagnosis. However, it has a sensitivity of only 20%, whereas other tests such as polymerase chain reaction have a sensitivity of 63%; histological examination has 65% sensitivity, and mycobacterium culture has 80% sensitivity [5].

Around one-third of patients with wrist tuberculosis have a concomitant pulmonary infection, with two-thirds occurring in isolation [6]. Plain radiography of the wrist and chest is needed to establish the diagnosis appropriately. Typically, plain radiography shows sclerosis and osteolytic lesions in the bones involved. This is nonspecific and can be present in conditions such as pyogenic osteomyelitis, inflammatory arthritis, and malignancy [7]. Sometimes, this condition can be misleading as patients also have bone destruction similar to that which can be found in that particular disease, whereas chronic bone infection should have sequestrum, involucrum, and cloaca in the radiograph six weeks or more following acute osteomyelitis [8]. CT and MRI scans are also nonspecific. However, they are useful in determining the extent of the swelling. In this case, we used ultrasound to evaluate the extent of the swelling since the consistency was fluctuant and there was purulent discharge on wrist movement.

The diagnosis can be confirmed by recognition of Mycobacterium tuberculosis in cultures and a histological pattern that is typical for tuberculosis. This is usually described as necrotizing granulomatous inflammation composed of epithelioid histiocytes surrounding a central necrotic zone with multinucleated giant cells and lymphocytes [1].

Treatment of tuberculosis is generally non-operative and involves a combination of anti-tuberculous medications that consist of a two-month-long intensive phase using a combination of rifampicin, isoniazid, ethambutol, and pyrazinamide followed by a continuation phase of rifampicin and isoniazid for another four months, as suggested by the World Health Organization in 2017 [9].

Most (around 75%) cases of tuberculosis of the wrist are successfully treated with anti-tuberculosis medications and would have good hand and wrist functional outcomes [10]. Nevertheless, surgery is recommended when there is nerve compression, impending bone collapse, or the need for joint debridement, drainage of a large abscess, or deformity correction in the setting of healed disease [5].

## Conclusions

Tuberculosis is still a major healthcare burden that needs medical professionals’ attention. Tuberculosis is not only confined to the pulmonary system, but extrapulmonary tuberculosis also exists. Even though tuberculosis of the wrist only accounts for a small proportion of extrapulmonary tuberculosis cases, one should be aware of this so that early diagnosis and treatment can be offered. Treatment mostly revolves around anti-tuberculosis medications alone, whereas surgery is reserved for select cases.

## References

[1] Mustafa Karakaplan, MD Muhammed Köroğlu, MD Zeynep Maraş Özdemir, MD Kadir Ertem, MD MD (2017). Isolated Tuberculosis of capitate and triquetrum. J Wrist Surg.

[2] Sivasamy P, Bajuri MY, Ghani AW (2020). Tuberculosis of the left wrist joint and spine. Cureus.

[3] Sharma SK, Mohan A (2004). Extrapulmonary tuberculosis. Indian J Med Res.

[4] Prakash J (2014). Tuberculosis of capitate bone in a skeletally immature patient: a case report. Malays Orthop J.

[5] Al-Qattan MM, Al-Namla A, Al-Thunayan A, Al-Omawi M (2011). Tuberculosis of the hand. J Hand Surg Am.

[6] Ramos RFM, Cancian L, Calcagnotto F, Zeni R, Varela G (2017). Synovial tuberculosis of the hand: an ancient disease in an unusual localization. Indian J Plast Surg.

[7] Spiegel DA, Singh GK, Banskota AK (2005). Tuberculosis of the musculoskeletal system. Tech Orthop.

[8] Chuah SK, Bajuri MY, Mohd Nor F (2020). Chronic osteomyelitis revisited: a case report. Cureus.

[9] (2020). Guidelines for treatment of drug-susceptible tuberculosis and patient care (2017 update). https://www.who.int/tb/publications/2017/dstb_guidance_2017/en/.

[10] Altayeb Mussa M, Fitzgerald O’Connor E, Waterston S, Taylor M, Iwuagwu F (2013). Isolated tuberculosis of the wrist: a rare case of extrapulmonary tuberculosis. Intl J Case Rep Images.

